# Septum Pellucidum Cavernous Malformation: A rare entity

**DOI:** 10.18295/squmj.3.2024.019

**Published:** 2024-08-29

**Authors:** Asma AlHatmi, Rajeev Kariyattil, Eiman Al-Ajmi

**Affiliations:** 1 Department of Radiology & Molecular Imaging, Sultan Qaboos University Hospital, Sultan Qaboos University, Muscat, Oman; 2 Department of Surgery, Sultan Qaboos University Hospital, Sultan Qaboos University, Muscat, Oman

A 30-year-old male patient with no medical history presented in 2022 to the emergency department of a tertiary care hospital in Muscat, Oman, with a 1-week history of persistent headaches and intermittent vomiting. He had no history of seizures, visual disturbances, neurological deficits or loss of consciousness. On examination, he was fully conscious and alert, with an unremarkable neurological assessment. Ophthalmological examination revealed no evidence of papilloedema.

A computed tomography (CT) scan showed a well-defined hyperdense lesion in the septum pellucidum, bulging bilaterally into the lateral ventricles, with minimal dilation of the ventricles and no signs of acute hydrocephalus. Subsequent magnetic resonance imaging (MRI) of the brain revealed a well-circumscribed lesion in the septum pellucidum, characterised by heterogeneous signal intensity, a hypointense hemosiderin rim and an internal bubbly appearance, forming a ‘popcorn’ pattern on T2-weighted images. Significant blooming was observed on T2* gradient echo images at the lesion site, indicative of blood products [Figure 1]. There was no enhancement of the lesion, surrounding oedema, significant mass effect or intraventricular haemorrhage. These imaging findings were in keeping with a diagnosis of septum pellucidum cavernous malformation.

Given the absence of clinical evidence of raised intracranial pressure (ICP) or radiological signs of acute bleeding or ventricular obstruction, the patient was managed conservatively. He remained asymptomatic and a follow-up MRI after 18 months showed an interval reduction in the size of the lesion, with a siderotic rim and internal old blood products [Figure 2]. The patient will undergo annual MRIs of the brain for next 2 years and has been instructed to promptly report any new symptoms.

We present this case of septum pellucidum cavernoma, discussing its imaging features, differential diagnosis and treatment options. Septum pellucidum cavernoma is an extremely rare entity, with only a few similar cases reported in the literature.

Informed written consent for publication was obtained from the patient.

## Comment

Cerebral cavernous malformations, or cavernomas, are a type of vascular malformations in the central nervous system (CNS), with a prevalence of 0.02–13%. Among CNS vascular malformations, they are the third most common, following developmental venous anomalies (DVA) and capillary telangiectasias, accounting for 5–13% of cases.^1^^,^^2^ Cavernomas can be sporadic or familial, with major risk factors for acquiring cavernomas including a positive family history, prior radiotherapy or head trauma.^1^^,^^3^ Cavernomas may be solitary or multiple, particularly in hereditary types and are often associated with other vascular malformations, most commonly DVAs.^1^ Approximately 50% of CNS cavernomas are incidental findings. They exhibit a wide range of clinical presentations depending on their location, size and associated complications such as mass effect or haemorrhage.^2^^,^^4^ Between 74–90% of cerebral cavernomas are located in the supratentorial region, where they are commonly associated with headaches, seizures and focal neurological deficits, while infratentorial cavernomas are more frequently associated with bleeding.^1^^,^^2^

Intraventricular cavernomas are rare, occurring in less than 10% of cases.^5^ Septum pellucidum cavernomas are an extremely rare subtype of intraventricular cavernoma.^1^^,^^2^ The septum pellucidum is a thin midline anatomical sheet located between the anterior horns of the lateral ventricles, extending from the corpus callosum to the fornix, an important structure connecting the hippocampus to other parts involved in memory function.^1^ Bleeding within the septum pellucidum cavernoma can damage the fornix, leading to long-term memory impairment and anterograde amnesia.^1^^,^^4^ Septum pellucidum cavernomas can also extend into either side of the lateral ventricle, potentially causing hydrocephalus by compressing the foramen of Monro.^4^ Haque *et al*. published a review article and descriptive analysis of septum pellucidum cavernomas, which included 10 cases reported in the English literature.^1^ The median age at diagnosis was 42 years, with a majority of patients being male. Headache was the most common presenting symptom, seen in 70% of cases, while 20% presented with impaired memory. One-third of the patients included in the study presented with complications such as haemorrhage and hydrocephalus.^1^

Neuroimaging plays a crucial role in diagnosing cavernomas. Large cavernomas appear as hyperdense lesions on CT scans, while small cavernomas can be difficult to visualise on CT.^4^ MRI, however, provides superior assessment, showing the characteristic ‘popcorn’ or ‘berry’ appearance with a rim of hemosiderin, as seen in the current patient. The signal intensity of cavernomas varies depending on the age of the blood products. Recent bleeds may be accompanied by surrounding oedema. T2* gradient echo images or susceptibility-weighted imaging (SWI) are the best sequences for detecting small cavernomas, particularly in hereditary cases. The enhancement of cavernomas can range from none to moderate.^1^^,^^2^ On digital subtraction angiography, cavernomas are occult lesions due to the lack of arteriovenous shunts.^1^

The differential diagnosis for septum pellucidum cavernomas includes haematoma, arteriovenous malformation, colloid cyst, neurocysticercosis or other neoplastic lesions, especially when associated with haemorrhage or calcifications, such as central neurocytoma, ependymoma and subependymal giant cell astrocytoma.^1^^,^^2^^,^^5^

The management of CNS cavernomas depends on their location and symptoms. Asymptomatic lesions can be managed conservatively. Symptomatic lesions causing mass effect, epilepsy, focal neurological deficit or repeated haemorrhages require surgical intervention. Cavernomas of the septum pellucidum with established or imminent ventricular obstruction warrant prompt surgical intervention. Various surgical approaches have been reported, with the most common being the transcranial transcallosal anterior interhemispheric approach.^1^^,^^2^^,^^4^ Although neuroendoscopy is a minimally invasive procedure with fewer complications, its role in excising septum pellucidum cavernomas is questionable due to the potential difficulty in controlling bleeding.^1^ The role of radiotherapy is debatable and is sometimes used for symptomatic control of certain intraparenchymal cavernomas.^5^ Some studies suggest that radiation increases the risk of cavernoma bleeding, interval growth and recurrence.^1^^,^^3^ Complete surgical resection of septum pellucidum cavernomas is curative, leading to clinical improvement and no recurrence.^1^^,^^3^ However, a few cases reported persistent memory impairment that was present at the time of diagnosis, likely due to irreversible damage to the fornix.^1^^,^^3^

## Figures and Tables

**Figure 1:**
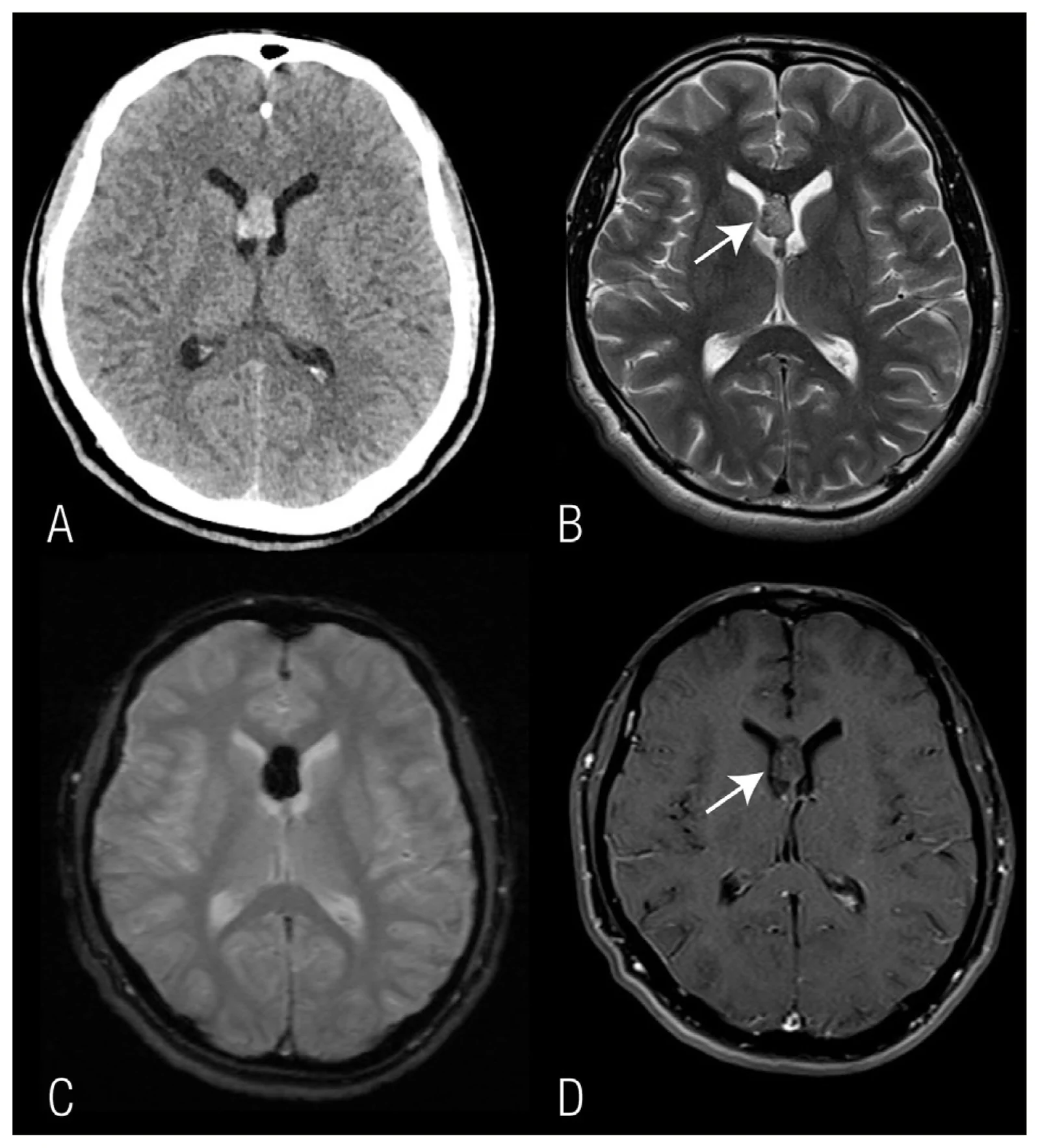
**A**: Axial non-enhanced computed tomography image showing a hyperdense lesion of the septum pellucidum at the level of the frontal horns, bulging bilaterally into the lateral ventricles. **B**: Axial T2-weighted image showing the lesion to be T2 hyperintense with a bubbly appearance. **C**: T2* gradient echo image showing significant blooming at the site of the lesion. **D**: Post-contrast axial T1-weighted image showing no significant enhancement.

**Figure 2:**
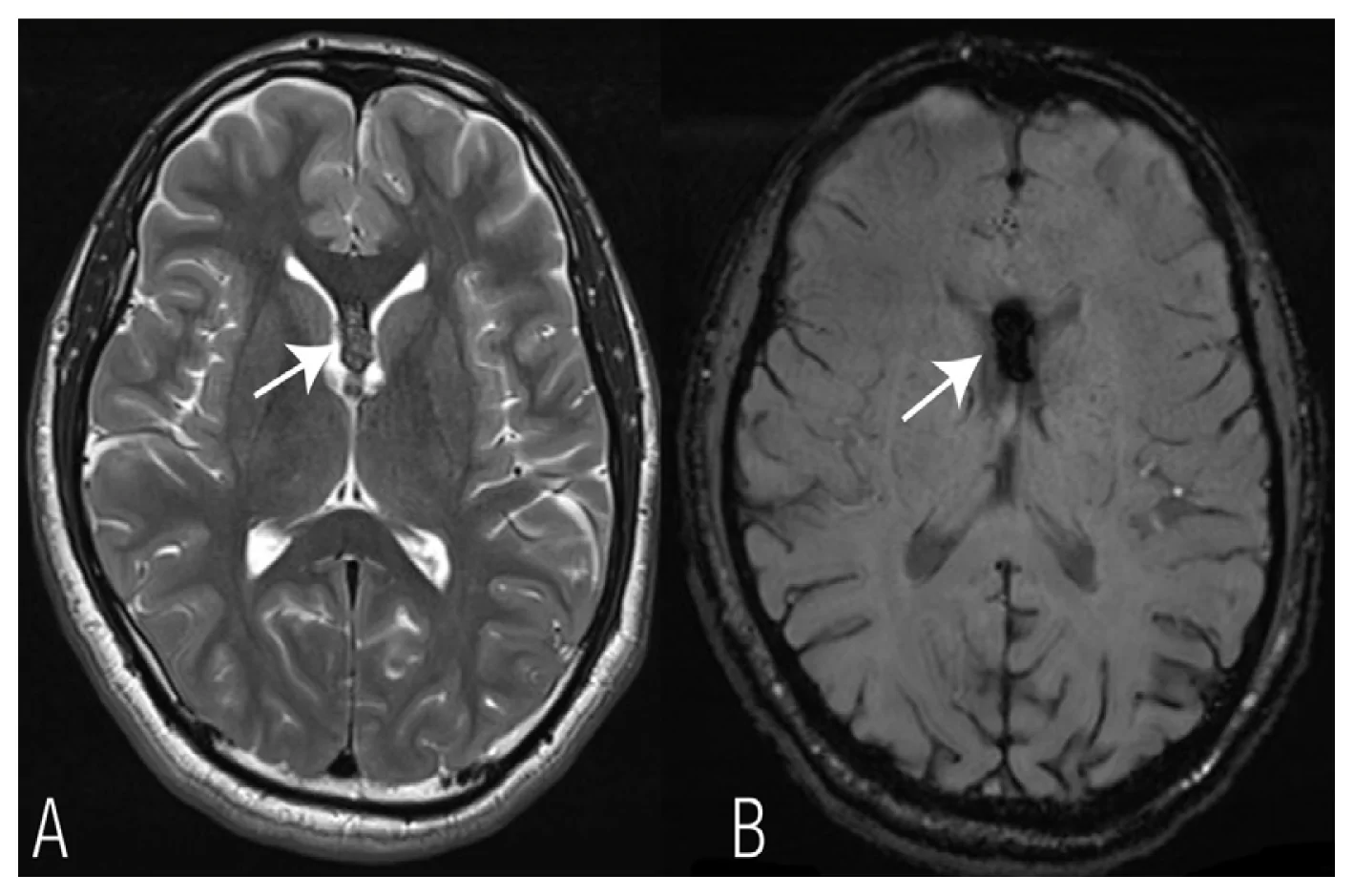
Magnetic resonance imaging at follow-up after 18 months showing (**A**) an interval reduction in the size of the lesion with a siderotic rim on an axial T2-weighted image (arrow) and (**B**) susceptibility effects due to old blood products in susceptibility-weighted imaging (arrow).

## References

[1] Haque S, Islam A, Rahman T, Hossain MD, Siddik AB, Shourav MMI (2021). Cavernoma in septum pellucidum: descriptive analysis and review of existing literature. J Cancer Sci Clin Ther.

[2] Paiva A, Lovato RM, Araujo JO, Veiga J (2020). Septum Pellucidum Cavernoma: A Case Report and Anatomical Consideration of an Extremely Rare Lesion. Turk Neurosurg.

[3] Picolas C, Faropoulos K, Kekempanou K, Gatzounis G (2016). Case Report of a Septum Pellucidum Cavernoma Surgically Resected via Inferior Parietal Approach and Short Literature Review. Open J Mod Neurosurg.

[4] Muzumdar D, Avinash KM, Ramdasi R (2015). Cavernoma of the septum pellucidum in the region of foramen of Monro. Neurol India.

[5] Faropoulos K, Panagiotopoulos V, Partheni M, Tzortzidis F, Konstantinou D (2015). Therapeutic management of intraventricular cavernoma: case series and review of the literature. J Neurol Surg A Cent Eur Neurosurg.

